# Adaptive Estimation of Multiple Fading Factors for GPS/INS Integrated Navigation Systems

**DOI:** 10.3390/s17061254

**Published:** 2017-06-01

**Authors:** Chen Jiang, Shu-Bi Zhang, Qiu-Zhao Zhang

**Affiliations:** 1 School of Environment Science and Spatial Informatics, China University of Mining and Technology, Xuzhou 221116, China; jiangchen@cumt.edu.cn (C.J.); qiuzhao.zhang@cumt.edu.cn (Q.-Z.Z.); 2 Collaborative Innovation Center for Resource Utilization and Ecological Restoration of Old Industrial Base, China University of Mining and Technology, Xuzhou 221116, China

**Keywords:** cubature Kalman filter, integrated navigation, H-infinity filter, multiple fading filter, optimization

## Abstract

The Kalman filter has been widely applied in the field of dynamic navigation and positioning. However, its performance will be degraded in the presence of significant model errors and uncertain interferences. In the literature, the fading filter was proposed to control the influences of the model errors, and the H-infinity filter can be adopted to address the uncertainties by minimizing the estimation error in the worst case. In this paper, a new multiple fading factor, suitable for the Global Positioning System (GPS) and the Inertial Navigation System (INS) integrated navigation system, is proposed based on the optimization of the filter, and a comprehensive filtering algorithm is constructed by integrating the advantages of the H-infinity filter and the proposed multiple fading filter. Measurement data of the GPS/INS integrated navigation system are collected under actual conditions. Stability and robustness of the proposed filtering algorithm are tested with various experiments and contrastive analysis are performed with the measurement data. Results demonstrate that both the filter divergence and the influences of outliers are restrained effectively with the proposed filtering algorithm, and precision of the filtering results are improved simultaneously.

## 1. Introduction

With a full solution for position, velocity, and attitude, the GPS and INS systems are both effective navigation techniques which have found broad applications. GPS, characterized by high precision, is able to provide accurate navigation and positioning information. INS is a self-navigation system which needs not to send and receive signals. The GPS/INS integrated navigation system manifests great superiority by integrating the advantages of GPS and INS, and performance of the integrated system is better than that of either the GPS or INS system alone [1]. Thus, the integration of GPS and INS gives a more complete and accurate navigation solution, and this integrated system has been widely adopted in the field of dynamic navigation and positioning in recent years. The Kalman filter is the most popular real-time optimal estimator [2], and it has been commonly applied in navigation applications. In the GPS/INS integrated navigation system, the Kalman filter is also the most commonly used data fusion method [3,4,5]. The parameter estimates of the Kalman filter are optimal when the statistical information of the noise is correctly specified. However, the required information is difficult to achieve when the model errors have time varying characteristics [6,7], and the performance will be degraded with improper statistical information and outliers, and sometimes may even lead to a filter divergence [7]. Moreover, it is unavoidable, especially when caused by a complex perturbation in a signal degraded environment [3]. As one of the basic procedures for the quality control of the Kalman filter, the Detection, Identification and Adaptation (DIA) methods were proposed to eliminate the effects of outliers [8]. In the DIA methods, the outliers are detected and identified firstly, then deviations of the state estimates caused by the outliers are eliminated, finally, the adaptation is implemented in which the model is recovered from the identified errors. However, the identification is quite difficult, especially when the measurements are not accurate [9]. For instance, the detection and identification process are difficult to implement when the measurements contain multiple outliers or the continuously-changing outliers. Therefore, in order to weaken the influence of the outliers, it is essential to research the filtering and estimation algorithms which are more robust in the GPS/INS integrated navigation system [9,10,11].

To achieve a better performance with the GPS/INS integrated navigation system, the modeling and estimation problem should be addressed properly [7]. The former is concerned with developing better error models and the latter requires proper use of the estimation algorithm and the measurement information. In the Kalman filter, filter divergence often happens when big errors of the system model exist, due to the effects of the improper a prior information [12]. With a constant fading factor, the fading Kalman filter was proposed to overcome the shortcomings of the Kalman filter [13]. In recent years, research about the fading Kalman filter focuses on the estimation of the fading factor. An optimal adaptive algorithm with a single fading factor for fading Kalman filter was proposed [14], nevertheless, a single fading factor can hardly guarantee the optimization of the whole system model, and the filter divergence will be likely to happen when handling complex system model [15]. Various adaptive Kalman filters have been developed to achieve a better estimator [9,16,17,18]. Then the multiple fading filter [15,19] was proposed to improve the performance of the fading filter. In the field of navigation and positioning, an algorithm based on the magnitude of the predicted residuals was proposed to reduce the dynamic model errors [20]. However, most of the above algorithms show few abilities to control the influences of the uncertain interferences. Moreover, the GPS/INS integrated navigation is very likely to be affected by uncertainties due to the exogenous disturbances, device damage, and inaccurate sensor noise statistical information [10]. By minimizing the estimation error in the worst case, the H-infinity filtering algorithm provides an effective way to improve the robustness of the system model [21] and it can be used to address the uncertainties in the measurement noise [18,22].

This paper focuses on a comprehensive filtering algorithm for the GPS/INS integrated navigation system by combining the advantages of the multiple fading filter and the H-infinity filter. Based on the concept of fading memory and the optimization of the filter, a new multiple fading factor is proposed. Experiments are implemented with the data collected under actual conditions. Performances of different filtering algorithms are evaluated and the contrastive analysis are implemented. Results demonstrate that the new multiple fading filter algorithm shows a higher accuracy whether the abnormal measurements ever exist. In addition, the proposed fading filtering algorithm is easy to implement with a relatively reasonable computation burden.

The rest of this paper is organized as follows: in Section 2, the theory of the fading filter and the H-infinity filter are introduced, and the nonlinear form of the H-infinity filter is listed. In Section 3, a new multiple fading factor is proposed and a comprehensive filtering algorithm is constructed. In Section 4, rules of the GPS/INS integrated navigation system are introduced, and equations of the system for the loosely-coupled integrated navigation system are listed. Section 5 presents the advantages of the proposed filtering algorithm through actual experiments, and the results are discussed and analyzed. The conclusions are provided in Section 6.

## 2. Fading Filter and H-Infinity Filter

In this section, the basic rules of the fading filter are introduced and the recent fading factors are discussed, then the principles of the H-infinity filter and its nonlinear form are discussed.

### 2.1. Basic Rules of the Fading Filter

For the dynamic model equation and the measurement equation:

(1) $$\left\{ \begin{array}{l} x_{k}=\Phi_{k,k-1}x_{k-1}+w_{k}\\ z_{k}=H_{k}x_{k}+v_{k} \end{array}\right.,$$

the predicted state vector is presented as:

(2) $$x_{k/k-1}=\Phi_{k,k-1}x_{k-1},$$

where $x_{k}$ denotes the state vector at the epoch k, $\Phi_{k,k-1}$ denotes the state transition matrix, $H_{k}$ denotes the measurement matrix, $z_{k}$ denotes the measurement vector, $w_{k}$ and $v_{k}$ denote the state noise and measurement noise, respectively, and $x_{k/k-1}$ denotes the predicted state vector.

The recursion approach of the fading filter is given by:

(3) $$x_{k/k}=x_{k/k-1}+{\bar{K}}_{k}(z_{k}-H_{k}x_{k/k-1}),$$

(4) $${\bar{K}}_{k}={\bar{P}}_{k/k-1}H_{k}^{T}{\left(H_{k}{\bar{P}}_{k/k-1}H_{k}^{T}+R_{k}\right)}^{-1},$$

(5) $${\bar{P}}_{k/k-1}=S_{k}\Phi_{k,k-1}P_{k-1}{\Phi^{T}}_{k,k-1}+Q_{k},$$

where ${\bar{K}}_{k}$ denotes the equivalent gain matrix, ${\bar{P}}_{k/k-1}$ denotes the equivalent covariance matrix of the state vector, $S_{k}$ denotes the fading factor and $S_{k}\geq 1$, $P_{k-1}$ denotes the covariance matrix of the state vector at epoch k−1, $R_{k}$ and $Q_{k}$ denote the covariance matrices of the measurement noise and the state noise, respectively.

In the standard Kalman filter, the covariance matrix of the state vector is given by:

(6) $$P_{k/k-1}=\Phi_{k,k-1}P_{k-1}{\Phi^{T}}_{k,k-1}+Q_{k}.$$

In the fading filter, the covariance matrix of the a prior state is inflated for $S_{k}$ times which degrades the efficiency of the past state information, and the recent measurement information is regarded more seriously [12]. Obviously, compared with the standard Kalman filter, the state errors from the previous epoch are well controlled.

### 2.2. The Recent Fading Factors

The key problem in the fading filter is to find a suitable fading factor. With the optimization considered, a fading filter algorithm was proposed [14] where two forms of fading factors were derived, namely, the steepest descent method and the one-step method. It is quite difficult to apply the former method due to its complex computations. In practice, the latter is more applicable which is derived by:

(7) $$S_{k}=\max\{1,\frac{1}{n}tr(N_{k}M_{k}^{-1})\},$$

where:

(8) $$M_{k}=H_{k}\Phi_{k,k-1}P_{k-1}\Phi_{k,k-1}^{T}H_{k}^{T},$$

(9) $$N_{k}=P_{V_{k}}-H_{k}Q_{k}H_{k}^{T}-R_{k},$$

(10) $$P_{V_{k}}=E(V_{k}V_{k}^{T}),$$

(11) $$V_{k}=H_{k}x_{k/k-1}-z_{k},$$

where tr(⋅) denotes taking trace of a matrix, $V_{k}$ denotes the predicted residual vector, and $P_{V_{k}}$ denotes the covariance matrix of $V_{k}$, and ${\hat{P}}_{V_{k}}=\frac{1}{k}\sum_{i=1}^{k}V_{i}V_{i}^{T}$ [12]. Then Xia [14] proposed a simplified method, namely:

(12) $$S_{k}=\max\{1,tr(N_{k})/tr(M_{k})\},$$

the fading factor will increase along with $V_{k}$, and $S_{k}$ calculated in this way is optimal in theory.

A multiple fading filter algorithm was proposed by Geng [19], and the fading factor is given by:

(13) $$S_{k}=\mathrm{diag}(s_{1},s_{2},...,s_{t},1,...,1,...,1),$$

(14) $$s_{i}=\max\left(1,\sqrt{\frac{{\left[v_{i}(k)\right]}^{2}}{\lambda_{i}^{2}j_{ii}(k)\varepsilon_{i}}-\frac{b_{ii}(k)}{j_{ii}(k)}}\right),\;(i=1,2,...,t),$$

where $v_{i}(k)$ is the i−th element of $V_{k}$, t is the dimension of the measurement equation, $\lambda_{i}$ is the i−th observable element of $H_{k}$, $b_{ii}(k)$ is the i−th diagonal element of the matrix $B_{k}$ and $B_{k}=H_{k}Q_{k-1}H_{k}^{T}+R_{k}$, $j_{ii}(k)$ is the i−th diagonal element of the matrix $J_{k}$ and $J_{k}=\Phi_{k}P_{k-1}\Phi_{k}^{T}$, $\varepsilon_{i}$ is the threshold value fixed according to the Chi-square distribution at the given confident level. In this approach, only $\left(s_{1},s_{2},...,s_{t}\right)$ can be estimated adaptively due to their observabilities, and the other elements should be fixed as one.

### 2.3. Principles of the H-Infinity Filter

Assume that the dynamic model equation and the measurement equation is given by:

(15) $$\left\{ \begin{array}{l} x_{k}=\Phi_{k,k-1}x_{k-1}+w_{k}\\ y_{k}=L_{k}x_{k}\\ z_{k}=H_{k}x_{k}+v_{k} \end{array}\right.,$$

where $y_{k}$ denotes the state vector to be estimated, $L_{k}$ denotes the given matrix, and $L_{k}$ is often fixed by an identity matrix. No statistical information of the noises is assumed before the filtering.

The cost function J is defined by [21]:

(16) $$J=\frac{\sum_{k=1}^{N}{\left\|x_{k}-{\hat{x}}_{k}\right\|}^{2}}{{\left\|x_{0}-{\hat{x}}_{0}\right\|}_{P_{0}^{-1}}^{2}+\sum_{k=1}^{N}\left({\left\|w_{k}\right\|}_{Q_{k}^{-1}}^{2}+{\left\|v_{k}\right\|}_{R_{k}^{-1}}^{2}\right)},$$

where N denotes the total number of filtering time limit, $x_{0}$ denotes the initial value of the state vector x with the covariance matrix $P_{0}$, ${\hat{x}}_{0}$ and ${\hat{x}}_{k}$ denote the estimated state vectors of $x_{0}$ and $x_{k}$, respectively, and the expression ${\left\|x_{0}-{\hat{x}}_{0}\right\|}_{P_{0}^{-1}}^{2}$ denotes ${\left(x_{0}-{\hat{x}}_{0}\right)}^{T}P_{0}^{-1}(x_{0}-{\hat{x}}_{0})$.

The estimate of the state vector should be achieved under the conditions of ${\hat{x}}_{k}=\arg\;\min{\left\|J\right\|}_{\infty }$. However, the closed-form approach of an optimal H-infinity filter can be achieved only under special conditions [23]. Accordingly, a suboptimal recursion algorithm was proposed through setting a threshold value γ which satisfied the following Riccati inequality [1]:

(17) $$P_{k}^{-1}+H_{k}^{T}H_{k}-\gamma^{2}L_{k}^{T}L_{k}>0,$$

where $P_{k}$ is the covariance matrix of $x_{k}$. Then, the recursion formulas of the H-infinity filter in the linear systems, which are different with the standard Kalman filter, are obtained [24]:

(18) $$P_{k/k}=P_{k/k-1}-\Phi_{k/k-1}P_{k/k-1}[H_{k}^{T}\;L_{k}^{T}]R_{e,k}^{-1}\left[\begin{array}{l} H_{k}\\ L_{k} \end{array}\right]P_{k/k-1}\Phi_{k/k-1}^{T},$$

(19) $$R_{e,k}^{-1}=\left[\begin{array}{cc} I & 0\\ 0 & -\gamma^{2}I \end{array}\right]+\left[\begin{array}{l} H_{k}\\ L_{k} \end{array}\right]P_{k/k-1}[H_{k}^{T}\;L_{k}^{T}].$$

It should be noticed that the robustness of the filter is related to the threshold value γ, and the filter becomes more robust when γ decreases. However, it may cause a filter divergence if γ is too close to zero [23].

## 3. Adaptive Estimation of the Multiple Fading Factor

In this section, the adaptive estimation of the new multiple fading factor is proposed, and a multiple fading filtering algorithm is constructed with the proposed multiple fading factor. Finally, the recursion formulas of the new algorithm are derived.

As mentioned above, the performance of the Kalman filter may degrade due to the past information. A constant fading factor was thus designed to address this problem [14]. However, a constant fading factor cannot guarantee optimal filtering absolutely. A multiple fading factor was proposed to improve the performance of a single fading factor [19], whereas it is unsatisfactory with respect to the robustness of the filtering. Accordingly, a new multiple fading filter is proposed to further improve the performance of the fading filter.

For the a prior covariance matrix in the multiple fading filter:

(20) $$P_{k/k-1}=S_{k}\Phi_{k,k-1}P_{k-1}{\Phi^{T}}_{k,k-1}+Q_{k},$$

$S_{k}=\mathrm{diag}(s_{1},s_{2},...,s_{t},1,1,...,1)$. To keep the symmetry of $P_{k/k-1}$, the Equation (20) can be rewritten as:

(21) $$P_{k/k-1}=S_{k}\Phi_{k,k-1}P_{k-1}{\Phi^{T}}_{k,k-1}S_{k}^{T}+Q_{k}.$$

Based on the observability and the optimal fading factor, a new multiple fading factor is constructed as:

(22) $$s_{i}=\left\{ \begin{array}{ll} \max\{1,[N_{k}(i,i)]/[M_{k}(i,i)]\}, & i=1,2,...,t\\ 1 & ,\;\mathrm{other} \end{array}\right.,$$

where $N_{k}(i,i)$ and $M_{k}(i,i)$ represent the i−th diagonal elements of $N_{k}$ and $M_{k}$, respectively. Similarly, only $\left(s_{1},s_{2},...,s_{t}\right)$ can be estimated adaptively due to their observabilities, and the other elements should be fixed as one. The multiple fading factors in Equation (22) are constructed based on the optimal single fading factor in Equation (12) and they can guarantee the optimization of different data channels, thus the optimization of the whole filter is guaranteed.

Then, a new multiple fading H-infinity filtering algorithm is constructed. With the H-infinity filter adopted, the recursion formulas of the new algorithm for a linear system are listed below (the predicted state vector is calculated in the same way as Equation (2)):

(23) $${\bar{P}}_{k/k-1}=S_{k}\Phi_{k,k-1}P_{k-1/k-1}\Phi_{k,k-1}^{T}S_{k}^{T}+Q_{k},$$

(24) $$x_{k/k}=x_{k/k-1}+{\bar{K}}_{k}(z_{k}-H_{k}x_{k/k-1}),$$

(25) $${\bar{K}}_{k}={\bar{P}}_{k/k-1}H_{k}^{T}{\left(H_{k}{\bar{P}}_{k/k-1}H_{k}^{T}+R_{k}\right)}^{-1},$$

(26) $${\bar{P}}_{k/k}={\bar{P}}_{k/k-1}-\Phi_{k/k-1}{\bar{P}}_{k/k-1}[H_{k}^{T}\;L_{k}^{T}]{\bar{R}}_{e,k}^{-1}\left[\begin{array}{l} H_{k}\\ L_{k} \end{array}\right]{\bar{P}}_{k/k-1}\Phi_{k/k-1}^{T},$$

(27) $${\bar{R}}_{e,k}^{-1}=\left[\begin{array}{cc} I & 0\\ 0 & -\gamma^{2}I \end{array}\right]+\left[\begin{array}{l} H_{k}\\ L_{k} \end{array}\right]{\bar{P}}_{k/k-1}[H_{k}^{T}\;L_{k}^{T}],$$

where ${\bar{P}}_{k/k}$ denotes the a posteriori covariance matrix of the state, and $S_{k}$ is fixed through (22).

## 4. The GPS/INS Integrated Navigation System

In this section, the dynamic model and the state estimator are listed. Then the loosely-coupled GPS/INS integrated navigation system is discussed in detail.

In recent years, three main forms of integration are developed: the loosely-coupled, the tightly-coupled and the deeply-coupled. Compared with the other two forms of integration, the loosely-coupled navigation system is easier to be implemented and the computation process is more concise [25,26].

A 15-dimension state vector is designed in the loosely-coupled GPS/INS integrated navigation system in this paper. And the parameters denote the deviations of position, velocities, attitudes, and the random bias of the gyroscope and accelerometer, respectively. The state vector $\hat{X}$ is given by:

(28) $$\hat{X}=[\delta x,\delta y,\delta z,\delta v_{x},\delta v_{y},\delta v_{z},\delta \varphi_{e},\delta \varphi_{n},\delta \varphi_{u},\delta g_{x},\delta g_{y},\delta g_{z},\delta a_{x},\delta a_{y},\delta a_{z}].$$

The nonlinear differential error model for the INS system is defined by [6,27]:

(29) $$\left\{ \begin{array}{l} \Delta {\dot{R}}^{e}=\Delta V^{e}\\ \Delta {\dot{V}}^{e}=(I_{3\times 3}-C_{e^{\prime }}^{e})f^{e^{\prime }}+C_{b}^{e^{\prime }}\nabla^{b}-2\Omega_{ie}^{e}\Delta V^{e}\\ {\dot{\varphi }}^{e}=(I_{3\times 3}-C_{e}^{e^{\prime }})\omega_{ie}^{e}-C_{b}^{e^{\prime }}\varepsilon^{b}\\ {\dot{\nabla }}^{b}=0\\ {\dot{\varepsilon }}^{b}=0 \end{array}\right.,$$

where $\Delta R^{e}$ and $\Delta V^{e}$ are the position and velocity errors under the computer e frame, respectively, ${\dot{\varphi }}^{e}$ is the attitude error between e frame and the platform $e^{\prime }$ frame, $I_{3\times 3}$ is the identity matrix, $C_{e^{\prime }}^{e}$ is the rotation matrix between e and $e^{\prime }$ frame, $C_{b}^{e^{\prime }}$ is the rotation matrix between the body frame and $e^{\prime }$ frame, $\Omega_{ie}^{e}$ is the skew symmetric matrix of earth rotation rate $\omega_{ie}^{e}$, $\nabla^{b}$ and $\varepsilon^{b}$ are the gyroscope and accelerometer drift errors under the body frame, respectively.

In this paper, the cubature Kalman filter is adopted to address the nonlinear problem of the integrated navigation system. For a discrete nonlinear system:

(30) $$\left\{ \begin{array}{l} x_{k}=f(x_{k-1})+w_{k}\\ z_{k}=h(x_{k})+v_{k} \end{array}\right.,$$

where f(⋅) and h(⋅) denote the known nonlinear functions. The cubature points are generated by:

(31) $$\xi =\sqrt{\frac{m}{2}}{\left[1\right]}_{i},\;i=1,...,m,$$

where m denotes the number of the cubature points and m=2n, n denotes the dimension of the state vector, [1] denotes the integral and symmetrical set of points. Then equations of the cubature Kalman filter are given by [28].

*(i) Time update*

(32) $$\left\{ \begin{array}{l} s_{k-1/k-1}=SVD(P_{k-1/k-1})\\ X_{k-1,k-1}=s_{k-1/k-1}\xi +x_{k-1/k-1}\\ X_{k/k-1}^{*}=f(X_{k-1,k-1}) \end{array}\right.,$$

(33) $$x_{k/k-1}=\frac{1}{m}\sum_{i=1}^{m}X_{i,k/k-1}^{*},$$

(34) $$P_{k/k-1}=\frac{1}{m}\sum_{i=1}^{m}X_{i,k/k-1}^{*}X_{i,k/k-1}^{*T}-x_{k/k-1}x_{k/k-1}^{T}+Q_{k}.$$

*(ii) Measurement update*

(35) $$\left\{ \begin{array}{l} s_{k/k-1}=SVD(P_{k/k-1})\\ X_{k/k-1}=s_{k/k-1}\xi +x_{k/k-1}\\ Z_{k/k-1}=h(X_{i,k/k-1})\\ z_{k/k-1}=\frac{1}{m}\sum_{i=1}^{m}Z_{i,k/k-1}\\ P_{zz,k/k-1}=\frac{1}{m}\sum_{i=1}^{m}Z_{i,k/k-1}Z_{i,k/k-1}^{T}-z_{k/k-1}z_{k/k-1}^{T}+R_{k}\\ P_{xz,k/k-1}=\frac{1}{m}\sum_{i=1}^{m}X_{i,k/k-1}Z_{i,k/k-1}^{T}-x_{k/k-1}z_{k/k-1}^{T} \end{array}\right.,$$

then the final measurement update equations are obtained:

(36) $$K_{k}=P_{xz,k/k-1}P_{zz,k/k-1}^{-1},$$

(37) $$x_{k/k}=x_{k/k-1}+K_{k}(z_{k}-z_{k/k-1}),$$

(38) $$P_{k/k}=P_{k/k-1}-K_{k}P_{zz,k/k-1}K_{k}^{T},$$

where s denotes the square root of the covariance matrix P, $X_{k-1,k-1}$ denotes the cubature points of the states vector, $X_{k/k-1}^{*}$ denotes the propagated cubature points, SVD denotes the singular value decomposition of a matrix; $Z_{k/k-1}$ denotes the propagated cubature points of the measurement vector. Accordingly, the multiple fading filtering algorithm for the GPS/INS integrated navigation system is listed below:

*(i) Time update*

(39) $$\left\{ \begin{array}{l} x_{k/k-1}=\frac{1}{m}\sum_{i=1}^{m}X_{i,k/k-1}^{*}\\ P_{k/k-1}=S_{k}\left(\frac{1}{m}\sum_{i=1}^{m}X_{i,k/k-1}^{*}X_{i,k/k-1}^{*T}-x_{k/k-1}x_{k/k-1}^{T}\right)S_{k}^{T}+Q_{k} \end{array}\right.,$$

*(ii) Measurement update*

(40) $$\left\{ \begin{array}{l} x_{k/k}=x_{k/k-1}+K_{k}(z_{k}-z_{k/k-1})\\ K_{k}=P_{xz,k/k-1}P_{zz,k/k-1}^{-1}\\ P_{k/k}=P_{k/k-1}-[P_{xz,k/k-1}\;P_{k/k-1}]{\left[\begin{array}{cc} P_{zz,k/k-1}-R_{k}+I & P_{xz,k/k-1}^{T}\\ P_{xz,k/k-1} & P_{k/k-1}-\gamma^{2}I \end{array}\right]}^{-1}\left[\begin{array}{l} P_{xz,k/k-1}^{T}\\ P_{k/k-1}^{T} \end{array}\right] \end{array}\right..$$

In the loosely-coupled GPS/INS integrated navigation system, the differences of position and velocity between GPS and INS are regarded as the measurement inputs to the integration algorithm, namely:

(41) $$Z_{\rho }(t)=\rho_{\mathrm{GPS}}-\rho_{\mathrm{INS}},$$

where $\rho_{\mathrm{GPS}}$ and $\rho_{\mathrm{INS}}$ are the position and velocity information output by the GPS and INS, respectively. Then the measurement vector is presented as:

(42) $$Z_{k}=\left[\begin{array}{l} r_{\mathrm{GPS}}-r_{\mathrm{INS}}\\ v_{\mathrm{GPS}}-v_{\mathrm{INS}} \end{array}\right],$$

where $Z_{k}$ denotes the measurement vector of the integrated navigation system at epoch k, $r_{\mathrm{GPS}}$, $r_{\mathrm{INS}}$, $v_{\mathrm{GPS}}$, and $v_{\mathrm{INS}}$ are the position and velocity information of GPS and INS, respectively. In the loosely-coupled navigation system, the measurement equation is linear, then the Equation (40) can be rewritten as:

(43) $$\left\{ \begin{array}{l} x_{k/k}=x_{k/k-1}+K_{k}(z_{k}-H_{k}x_{k/k-1})\\ K_{k}=P_{k/k-1}H_{k}^{T}{\left(H_{k}P_{k/k-1}H_{k}^{T}+R_{k}\right)}^{-1}\\ P_{k/k}=P_{k/k-1}-[P_{k/k-1}H_{k}^{T}\;P_{k/k-1}]{\left[\begin{array}{ll} H_{k}P_{k/k-1}H_{k}^{T}+I & H_{k}P_{k/k-1}^{T}\\ P_{k/k-1}H_{k}^{T} & P_{k/k-1}-\gamma^{2}I \end{array}\right]}^{-1}\left[\begin{array}{l} H_{k}P_{k/k-1}^{T}\\ P_{k/k-1}^{T} \end{array}\right] \end{array}.\right.$$

Obviously, the observable variables are the position and velocity information of each direction, thus the corresponding fading factors can be estimated adaptively.

## 5. Testing

In this section, two cases of a loosely-coupled GPS/INS integrated navigation system are designed and implemented. Then the performance of the proposed filtering algorithm is discussed in comparison with the existing algorithms.

### 5.1. Experiment Schemes

To examine the performance of the proposed multiple fading filter, various actual experiments were performed. The data were obtained through a vehicle mounted GPS/INS integrated navigation system. In this system, two Trimble GPS receivers and a low cost inertial measurement unit (IMU) were adopted, and the main parameters of the IMU are listed in Table 1. 

**Figure 1:**
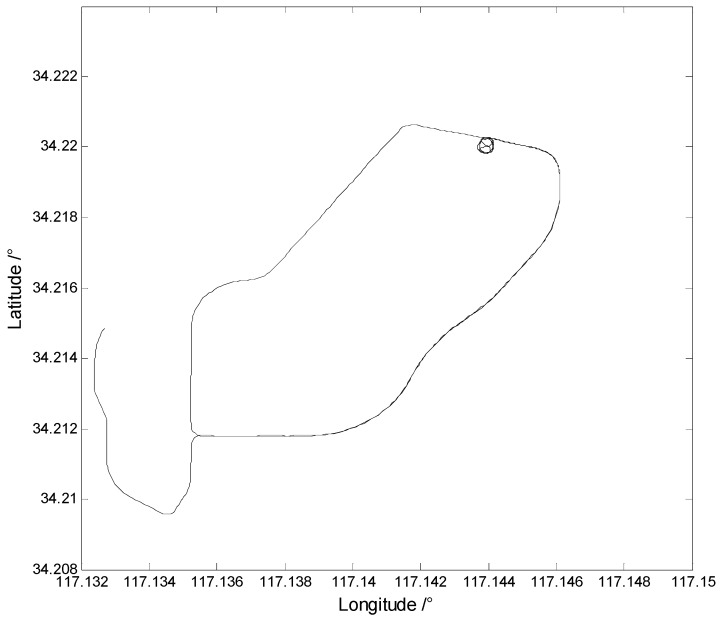
The motion trajectory of the land vehicle.

The update rate of GPS and IMU were set by 1 Hz and 100 Hz, respectively. The initial position and velocity errors are 1.0 m and 0.1 m/s, respectively. The initial position errors are set by 3.0 m, 3.0 m, 5.0 m, respectively, the initial velocity error is 0.5 m/s, and the initial alignment errors are set by 3°, 3°, 10°, respectively. Double difference GPS data were adopted with the position and velocity variances of 0.25 m^2^ and 0.0025 m^2^/s^2^, respectively. The precise results calculated by the double difference carrier phase were regarded as references. The motion trajectory of the land vehicle is displayed in Figure 1.

Then, two cases were designed to evaluate the stability and robustness of the new algorithm: the original measurements were adopted in case 1, and the measurements with perturbations in case 2. In each case, six schemes were performed:

Scheme 1: the cubature Kalman Filter (CKF);
Scheme 2: the H-infinity Filter (HF-CKF) (γ was set as 2);
Scheme 3: the conventional multiple fading filter (MF-CKF);
Scheme 4: the new multiple fading filter (NMF-CKF);
Scheme 5: the multiple fading H-infinity filter (MHF-CKF) (γ was set as 2);
Scheme 6: the new multiple fading H-infinity filter (NMHF-CKF) (γ was set as 2).

### 5.2. Results of the Experiments

**Figure 2:**
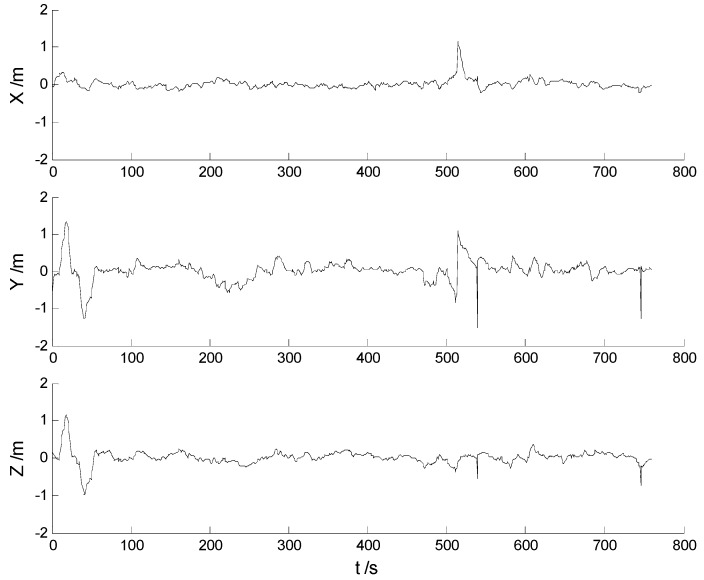
Position errors of the CKF algorithm.

**Figure 3:**
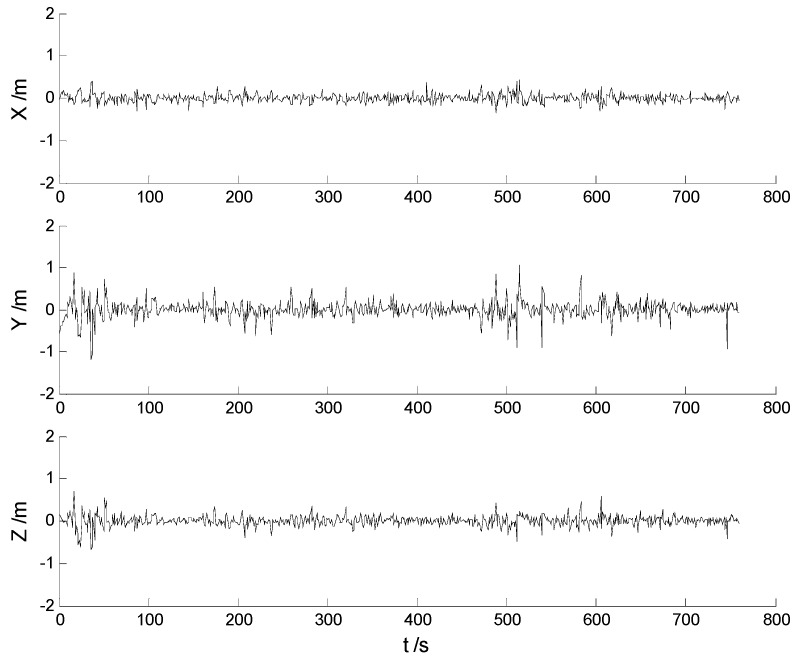
Position errors of the HF-CKF algorithm.

**Figure 4:**
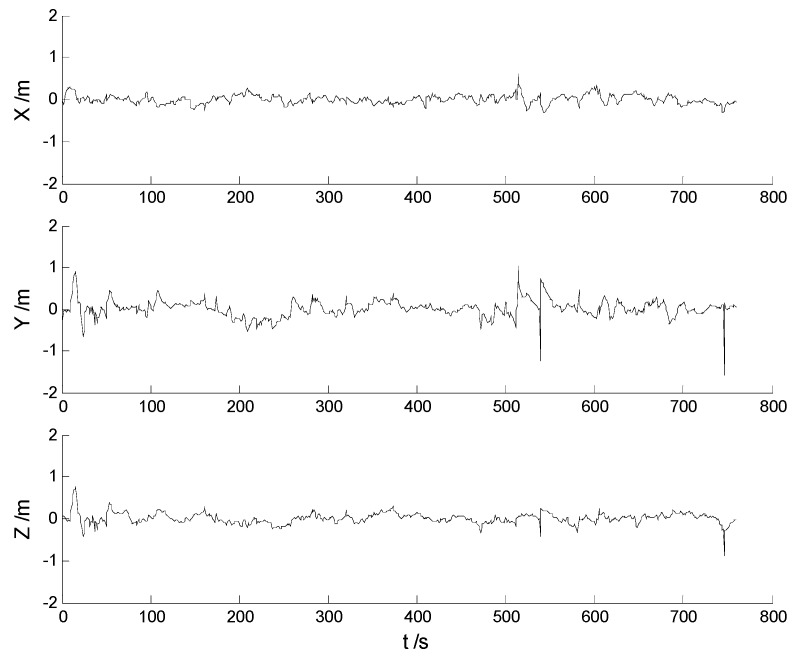
Position errors of the MF-CKF algorithm.

**Figure 5:**
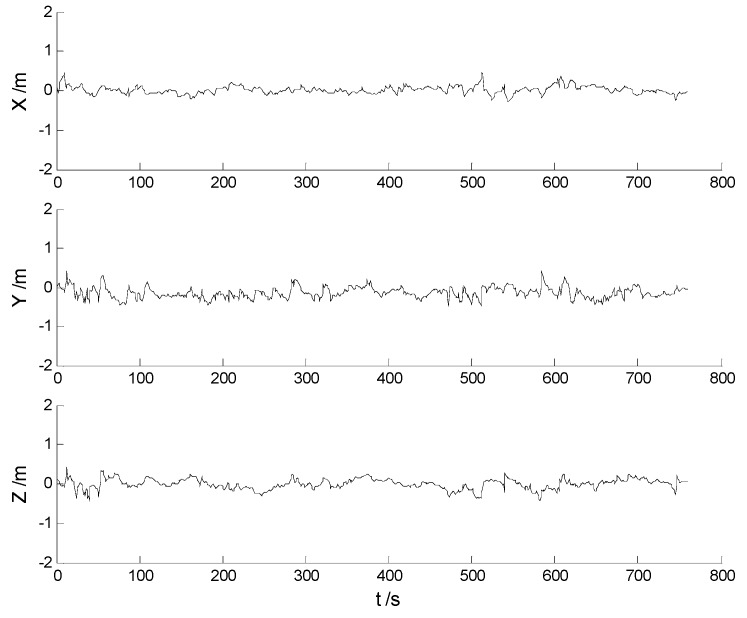
Position errors of the NMF-CKF algorithm.

**Figure 6:**
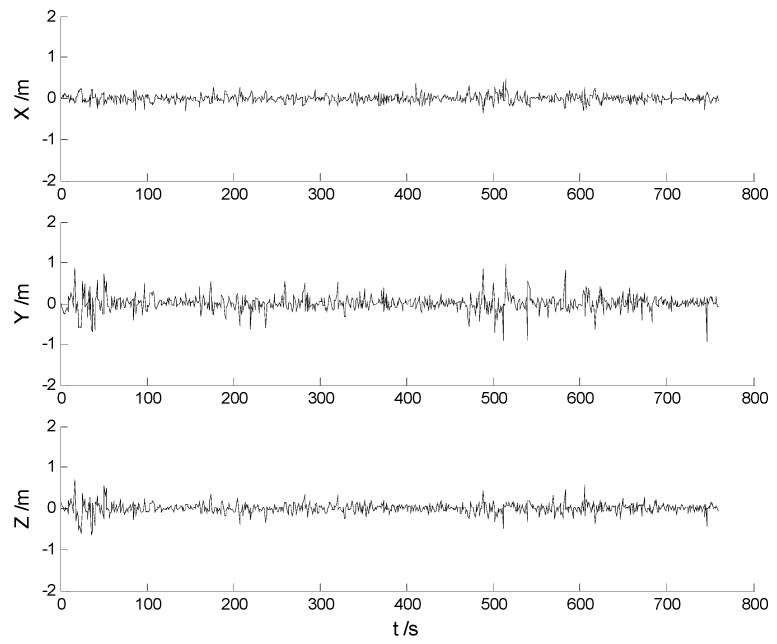
Position errors of the MHF-CKF algorithm.

**Figure 7:**
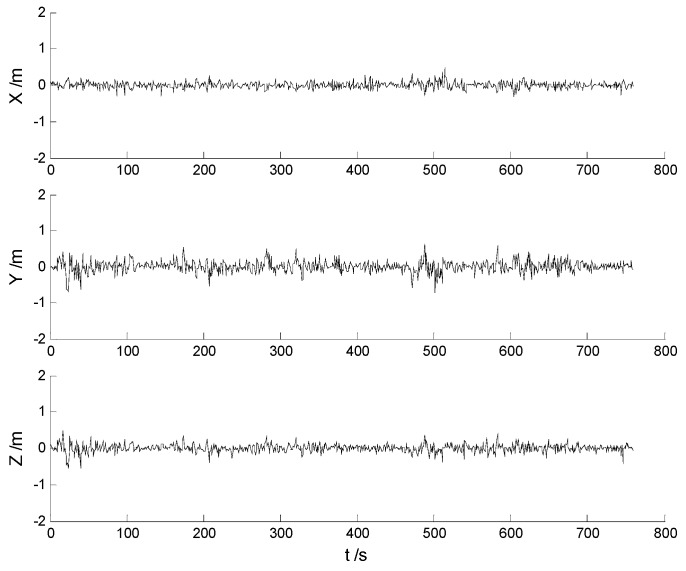
Position errors of the NMHF-CKF algorithm.

(1) Case 1:

In this case, each algorithm was performed with the original measurement data. The position errors in the X, Y, Z directions of the six schemes are demonstrated in Figure 2, Figure 3, Figure 4, Figure 5, Figure 6 and Figure 7:

The position and attitude RMSEs of the each scheme are listed in Table 2 and Table 3, respectively.

Big disturbances are found in Figure 2 which may occur when the vehicle is passing through the speed hump, and this indicates that the robustness of the CKF algorithm should be strengthened. It is concluded from Figure 2 and Figure 3 that, with the effects of the uncertainties controlled, the HF-CKF algorithm performs much better than the CKF algorithm. Compared with the CKF algorithm, Figure 4 and Figure 5 demonstrate that both the conventional and the new multiple fading filters manifest certain ability of controlling the influence of the model deviations. By integrating the advantages of the multiple fading filter and the H-infinity filter, the MHF-CKF and NMHF-CKF algorithms show better performance than the other algorithms which can be learned from the Figure 6 and Figure 7, and this indicates that the influences of the model deviations and uncertain interferences are well controlled with the MHF-CKF and NMHF-CKF algorithms. In Table 2, RMSEs of the MF-CKF and NMF-CKF algorithms are much smaller than those of the CKF algorithm, and it can be concluded that the fading factors have reduced the weight of the past state information. Consequently, compared with the conventional Kalman filter, the impacts of the state model errors are controlled effectively with the multiple fading filter algorithm, and thus a higher precision is obtained. Figure 7 and Table 2 demonstrate that the new multiple fading filter performs the best among the six schemes, and both the amplitudes of errors and the RMSEs are further reduced. As displayed in Table 3, there exists a little differences of the attitude errors between each algorithm, and the RMSEs of the NMHF-CKF algorithm are slightly smaller than those of the other algorithms.

**Figure 8:**
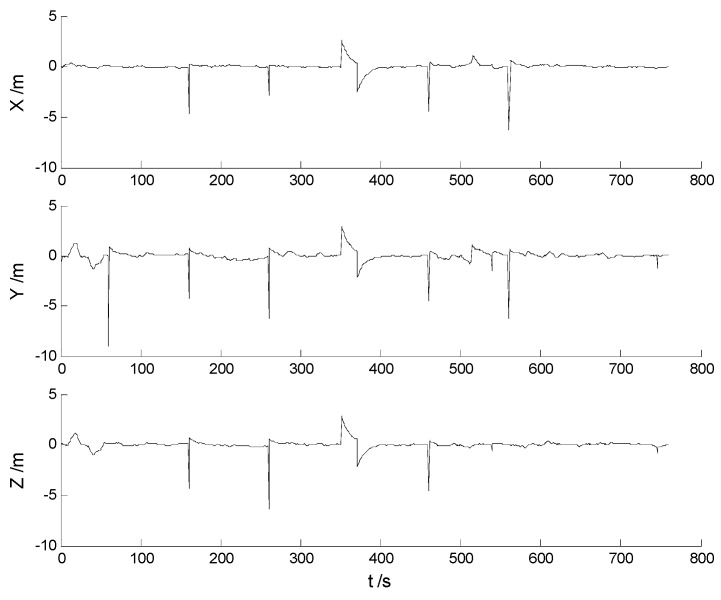
Position errors of the CKF algorithm.

**Figure 9:**
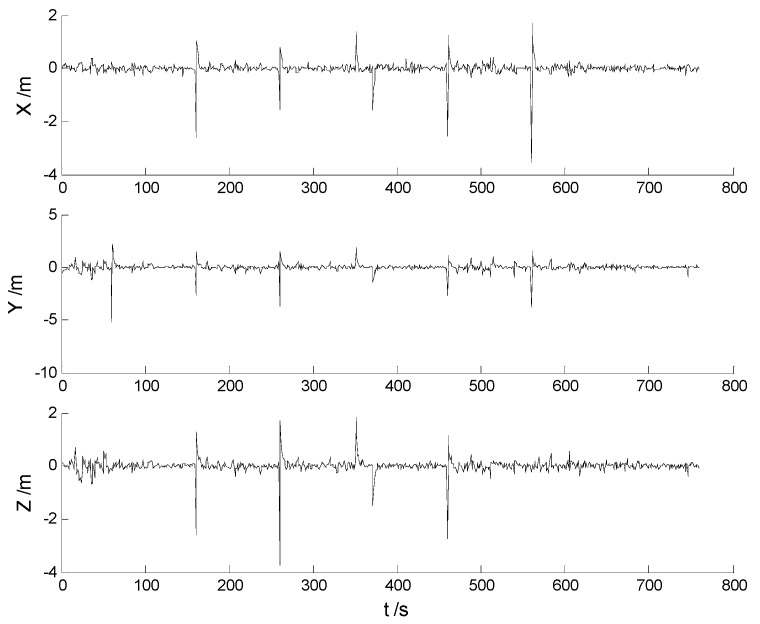
Position errors of the HF-CKF algorithm.

**Figure 10:**
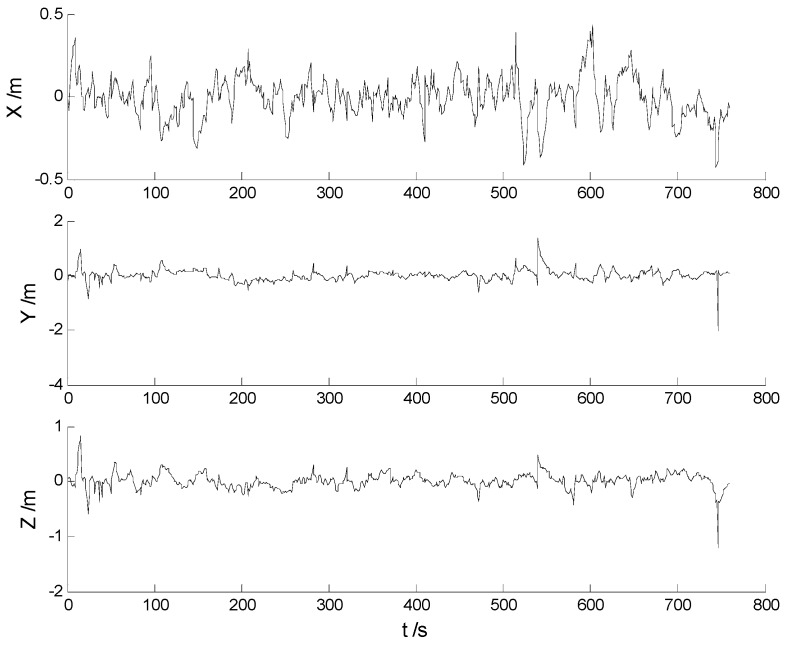
Position errors of the MF-CKF algorithm.

**Figure 11:**
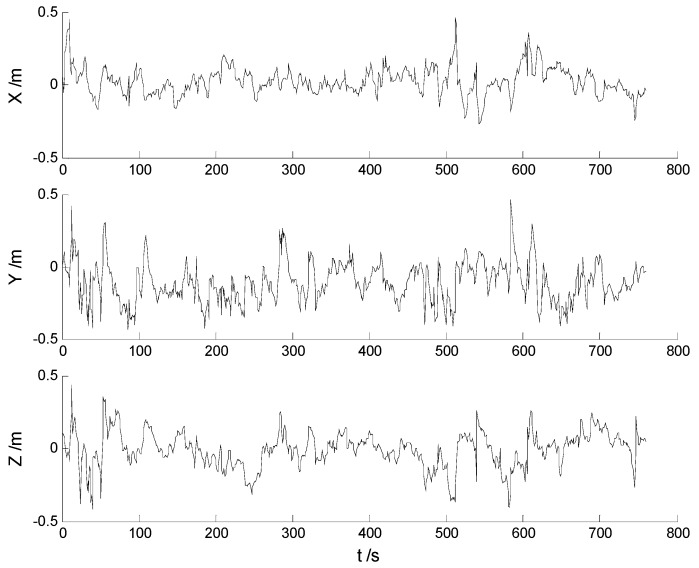
Position errors of the NMF-CKF algorithm.

**Figure 12:**
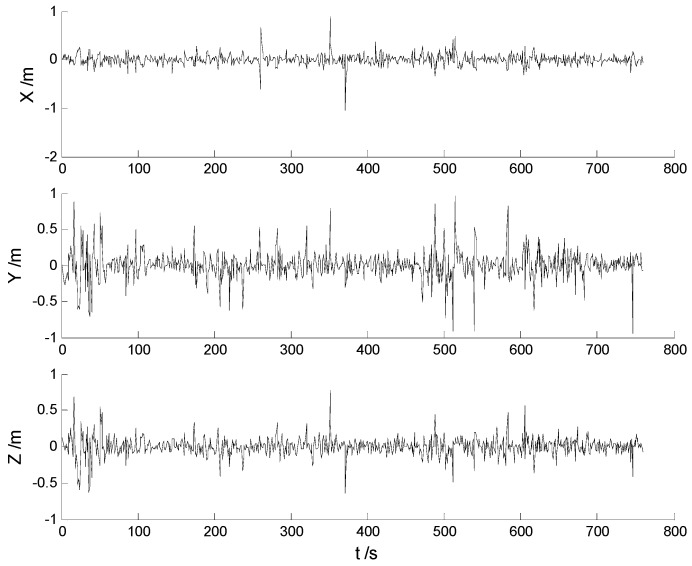
Position errors of the MHF-CKF algorithm.

**Figure 13:**
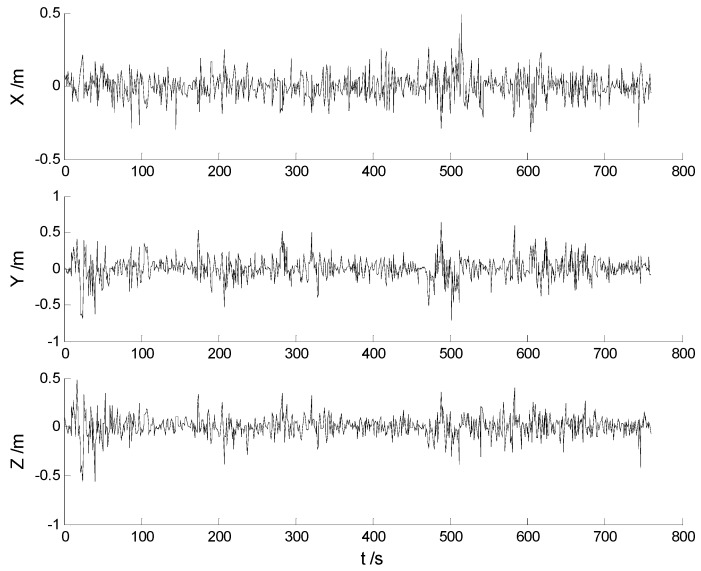
Position errors of the NMHF-CKF algorithm.

(2) Case 2:

In this case, ability of robustness for the above schemes is examined with both scattered and continuously-changing outliers. Thus the scattered outliers at the 160th, 260th, 360th, 460th, 560th epochs, respectively, and the continuously-changing outliers were added artificially to the GPS measurements at the 351th to the 380th epochs. Then the six schemes were implemented with the new data. Position errors of these schemes are displayed in Figure 8, Figure 9, Figure 10, Figure 11, Figure 12 and Figure 13:

In this case, the position errors are mainly caused by outliers. It is clearly demonstrated from the Figure 8 and Figure 9 that the CKF and HF-CKF algorithms show little ability to resist the outliers. On the contrary, both the conventional and the new fading filters manifest a noticeable robustness which can be seen from the Figure 10, Figure 11, Figure 12 and Figure 13. By integrating the advantages of the H-infinity filter, filtering precision is furtherly improved with the MHF-CKF and the NMHF-CKF algorithms. In addition, error amplitudes of the new multiple fading filter are smaller than those of the other filtering algorithms, which indicates that effects of both scattered and continuously-changing outliers are weakened effectively. Then the position and attitude RMSEs of each scheme are listed in Table 4 and Table 5:

In Table 4, position RMSEs of the CKF and the HF-CKF algorithms are significantly affected, and the HF-CKF algorithm exerts certain robustness compared with the CKF algorithm. Position RMSEs of the conventional and the new multiple fading filtering algorithms change little in the presence of outliers, and the new multiple fading filter shows a higher accuracy than the conventional multiple fading filter whether the H-infinity filter is ever adopted. The Table 5 indicates that the proposed algorithm performs better than the other algorithms, and the influence of the attitude errors are weakened further. Due to the fading factors, multiple data channels are adjusted depending on their observabilities of the state vector, and the state model errors from the previous epoch are well controlled, thus the filter becomes more stable and the filter divergence caused by the model deviations is restrained. It should be pointed out however, in practice, $N_{k}$ may sometimes become negative definite and $S_{k}$ has to be fixed by 1 now, and this indicates that the state model error may not be controlled effectively at the current epoch [12].

## 6. Conclusions

In this study, a new multiple fading factor is proposed based on the thought of fading memory and the optimization of the filter, and a comprehensive filtering algorithm is constructed by integrating the proposed multiple fading filter and the H-infinity filter. The new filtering algorithm is implemented in the loosely-coupled GPS/INS integrated navigation system. The detail conclusions in this paper are summarized below:

(1) The H-infinity filter based on the cubature Kalman filter performs better than the cubature Kalman filter, however, they are both affected significantly by the model errors and outliers. The conventional multiple fading filter shows certain robustness which can be furtherly enhanced.
(2) With the influences of the model deviations and the uncertain interferences controlled simultaneously, performance of the conventional multiple fading filter is further improved, and the higher precision is obtained by integrating the advantages of the multiple fading filter and the H-infinity filter. The proposed multiple fading filter is implemented in the loosely-coupled GPS/INS integrated navigation system, and the stability and robustness are demonstrated with the original data and the data with both the scattered and the continuously-changing outliers.

## Figures and Tables

**Table 1:** Main technological parameters of IMU and GPS.

Sensors	Random Bias	Random Constant Noise	RMSEs
			
Gyroscope	20 (°)/h	0.067 (°)/h^1/2^	-
Accelerometer	5 mg	50 μg/h^1/2^	-
GPS receiver	-	-	Position: 0.5 m; Velocity: 0.05 m/s

**Table 2:** Position RMSEs of each scheme (meter).

Axis	CKF	HF-CKF	MF-CKF	NMF-CKF	MHF-CKF	NMHF-CKF
X	0.129	0.096	0.108	0.093	0.095	0.088
Y	0.284	0.204	0.207	0.181	0.195	0.167
Z	0.188	0.128	0.127	0.121	0.126	0.112

**Table 3:** Attitude RMSEs of each scheme (degree).

Axis	CKF	HF-CKF	MF-CKF	NMF-CKF	MHF-CKF	NMHF-CKF
X	0.043	0.042	0.042	0.038	0.040	0.036
Y	0.051	0.049	0.052	0.042	0.048	0.040
Z	0.324	0.278	0.275	0.233	0.243	0.215

**Table 4:** Position RMSEs of each scheme (meter).

Axis	CKF	HF-CKF	MF-CKF	NMF-CKF	MHF-CKF	NMHF-CKF
X	0.482	0.257	0.124	0.109	0.114	0.092
Y	0.672	0.409	0.224	0.191	0.198	0.178
Z	0.488	0.273	0.140	0.127	0.130	0.121

**Table 5:** Attitude RMSEs of each scheme (degree).

Axis	CKF	HF-CKF	MF-CKF	NMF-CKF	MHF-CKF	NMHF-CKF
X	0.046	0.044	0.043	0.041	0.042	0.038
Y	0.052	0.050	0.056	0.048	0.050	0.046
Z	0.326	0.312	0.301	0.254	0.265	0.239
